# Selenium Yeast Alleviates Ochratoxin A-Induced Apoptosis and Oxidative Stress via Modulation of the PI3K/AKT and Nrf2/Keap1 Signaling Pathways in the Kidneys of Chickens

**DOI:** 10.1155/2020/4048706

**Published:** 2020-02-18

**Authors:** Kang Li, Zhongjun Cao, Yang Guo, Cui Tong, Shuhua Yang, Miao Long, Peng Li, Jianbin He

**Affiliations:** ^1^Key Laboratory of Zoonosis of Liaoning Province, College of Animal Science & Veterinary Medicine, Shenyang Agricultural University, Shenyang 110866, China; ^2^Tieling City Inspection and Testing and Certification Service Center (Animal Product Safety Testing Station), Tieling 112000, China

## Abstract

The purpose of this study was to investigate the protective effect and mechanism of yeast selenium (Se-Y) on ochratoxin- (OTA-) induced nephrotoxicity of chickens. A total of 80 one-day-old healthy chickens were randomly divided into 4 equal groups: control, OTA (50 *μ*g/kg OTA), Se-Y (0.4 mg/kg Se-Y), and OTA+Se-Y (50 *μ*g/kg OTA+0.4 mg/kg Se-Y). In the OTA chickens, differences in body weight, kidney coefficient, biochemical histological analysis, antioxidant capability, and the expression levels of the PI3K/AKT and Nrf2/Keap1 signaling pathway-related genes were observed. The levels of total superoxide dismutase (T-SOD), antioxidant capacity (T-AOC), catalase (CAT), and glutathione (T-GSH) significantly decreased, but the malondialdehyde (MDA) level of the kidneys significantly increased in the OTA treatment group. More importantly, treatment with Se-Y improved the antioxidant enzyme activities within the kidneys of chickens exposed to OTA. In addition, administration of OTA resulted in apoptosis and was associated with decreased expression of AKT, PI3K, and Bcl-2, which in turn enhanced expression of Caspase3, Bax, and P53. However, Se-Y improved the antioxidant defense system through activation of the Nrf2/Keap1 signaling pathway. Gene expression of Nrf2 and its target genes (HO-1, GSH-px, GLRX2, MnSOD, and CAT) was downregulated following OTA exposure. Conversely, Se-Y treatment resulted in a significant upregulation of the same genes. Besides, significant downregulations of protein expression of HO-1, CAT, MnSOD, Nrf2, and Bcl-2 and a significant upregulation of Caspase3 and Bax levels were observed after contaminated with OTA. Notably, OTA-induced apoptosis and oxidative damage in the kidney of chickens were reverted back to normal level in the OTA+Se-Y group. Taken together, the data suggest that Se-Y alleviates OTA-induced nephrotoxicity in chickens, possibly through the activation of the PI3K/AKT and Nrf2/Keap1 signaling pathways.

## 1. Introduction

Because of the omnipresent nature of fungi, mycotoxin contamination of animal feed poses a serious hazard to consumers and places a substantial burden on the health care system [1, 2]. Cereals (wheat, oats, barley) are among the food materials that are most frequently contaminated with mycotoxins. These cereals, which are used in the production of fermented foods, often contain ochratoxin (OTA), which results in consumption of contaminated cereal [3]. Mycotoxins are secondary metabolites, produced during fungal replication, that bioaccumulate in the contaminated feed. This has the potential to result in high levels of mycotoxins. The buildup of toxins in the cereal feed can then accumulate in the body fluids, organs and tissues, of the animals that consume the feed, potentially resulting in livestock and poultry mortality. However, exposure to OTA does not always result in death. With repeated sublethal exposures, the animals may develop immune dysfunction, which in turn degrades production performance. Of additional concern is the fact that OTA has been observed to accumulate and persist in meat, eggs, and milk which has the potential to endanger human health [4].

OTA is primarily produced by Aspergillus and Penicillium species of mold. Among the different OTAs, OTA type A is the most widespread and relevant fungal toxin [5]. In addition, OTA has been shown to suppress immune function and inhibit the intestinal absorption of nutrients, resulting in poor growth and disease resistance in livestock [6]. Contamination of feed with OTA can cause serious health problems and has been associated with several human and animal diseases, including endemic nephropathies and urinary tract tumors in humans, porcine nephropathy, and poultry ochratoxicosis [7]. Furthermore, OTA has been reported to initiate skin tumor development, which may be the result of oxidative stress and DNA damage caused by activation of the MAPK pathway [8]. Numerous studies have implicated OTA in nephropathies [9]. It is the agent that has been demonstrated to induce nephritis in chickens and is suspected to be a major etiological agent in Balkan endemic nephropathy (BEN) and urinary tract cancer [9, 10]. It has been reported that OTA-induced toxicity can activate apoptosis, in the nephrons of the kidneys [11]. Exposure of poultry to OTA is responsible for avian ochratoxicosis [12]; however, the sensitivity of different birds species to OTA is variable. In addition, the role of OTA in nephrotoxicity remains elusive. As contamination of poultry feed has potentially severe consequences for bird health, the poultry industry, and human health alike, a clear understanding of the effects of mycotoxins is critical.

Selenium (Se) is an essential trace mineral and a natural antidote to pollution as it is a constituent of the main antioxidative enzyme glutathione peroxidase (GSH-px). OTA is targeted to the kidney and causes nephrotoxicity. It has been reported that Se could alleviate porcine nephrotoxicity of OTA [13]. The use of appropriate Se supplement can enhance antioxidant and immune system functioning, thus promoting disease resistance [14, 15]. Recent studies have shown that, in animals and humans, Se can reduce the toxic effects of mycotoxins, including OTA, AFB1, T-2 toxin, and moniliformin [15]. In addition, recent studies have shown that the effects of hydroponically produced Se-enriched kale sprout (HPSeKS) and Se-Y on the bioavailability of selenium and the concentration of Se in whole eggs of laying hens are better than those of Se from a sodium selenite (SS) source [16, 17]. Moreover, it has been demonstrated that including appropriate Se-Y in the basal diet not only can aid in detoxification but can also promote muscle growth and improved muscle quality in chickens [18, 19]. Anti-OTA dietary supplements are of great interest due to the extensive toxicity of OTA and because many of these supplements do not cause significant feed safety problems without compromising nutrient loss. In recent years, there has been increasing interest in the use of Se-Y as an antioxidant feed supplement. The potential role of Se-Y to alleviate OTA-induced nephrotoxicity remains largely unknown.

Previous studies have shown that oxidative stress can be a key determinant of OTA-induced nephrotoxicity [20]. Oxidative stress can impede PI3K/AKT pathway signal transduction, leading to Nrf2 activation disorder, reduced nuclear translocation, and increased degradation through ubiquitination, resulting in decreased Nrf2 total protein levels. This ultimately results in downregulation of Nrf2 target gene expression [21]. The PI3K/AKT signaling pathway has been shown to play an important role in regulating cell growth, apoptosis, differentiation, and migration [22, 23]. As a downstream target of PI3K, AKT is activated by extracellular signals. Then, when AKT is activated through PI3K signaling, AKT initiates a series of intracellular responses, such as P53, Caspase3, Bcl-2, and Bax [24, 25]. Many of the effects associated with OTA appear to be mediated through oxidative stress. Numerous studies have implicated the importance of the nuclear factor E2-related factor 2 (Nrf2) in renal disorders [26, 27]. Increasing evidence has also shown that nuclear translocation of Nrf2 requires activation of the PI3K/AKT pathway [28, 29]. One of the downstream signaling proteins regulated by the PI3K/AKT pathway is Nrf2, which induces antioxidant enzyme responses, including HO-1, MnSOD, CAT, and GSH-px by activating the Nrf2/ARE pathway [30]. However, the precise mechanism through which the PI3K/AKT signaling pathway regulates Nrf2 activation in response to OTA in kidneys remains elusive. Therefore, in the current study, the relationship between various signaling molecules and Nrf2 was investigated. Based on the initial, albeit limited, understanding of the pathway mechanisms, it was hypothesized that Se-Y provides an effective protection in the development of OTA-induced renal injury.

Although the OTA toxicity has been well characterized, the protective efficacy of Se-Y against OTA-induced renal injury in chickens has not been explored. Dietary antioxidants have garnered significant attention in the recent years due to their antioxidant and therapeutic properties. Reducing oxidative stress is the key to reducing OTA toxicity. In view of the strong harmful effects of OTA, the development of effective strategies to prevent nephrotoxicity remains an urgent need for livestock and poultry health. In this study, we aimed to investigate the effects of Se-Y on OTA-induced nephrotoxicity of chickens.

## 2. Materials and Methods

### 2.1. Drugs and Chemicals

Standards of OTA were purchased from Pribolab (Immunos, Singapore; purity > 98%). Selenium yeast was provided by Angel Yeast (Hubei, China; purity > 99.5%). Pure OTA crystals were dissolved in absolute ethanol (1 mg/10 mL) and then mixed with 90 mL of sterile sunflower oil to prepare a suspension. All reagents in the experiment were of analytical grade.

### 2.2. Animals

A total of 80 one-day-old chickens were purchased from a commercial rearing farm (Shenyang Poultry Farm, Liaoning Province, China) and were allowed 3 days to acclimate to their new environment after transportation. All chickens were provided with water and animal feed, as well as the diets *ad libitum*. Each group of diets was prepared at the same time and stored in resealable bags before feeding. The experimental period lasted for 21 days. Animal experiments were conducted as approved by the Ethics Committee for Laboratory Animal Care (Animal Ethics Procedures and Guidelines of China) at the use of Shenyang Agricultural University (Permit No. 264SYXK<Liao>2011-0001, September 2018).

### 2.3. Experimental Design and Treatment

Each of the 80 one-day-old healthy broilers was randomly divided into 4 equal groups: (control, OTA (50 *μ*g/kg OTA, body weight), Se-Y (0.4 mg/kg Se-Y, diet), and OTA+Se-Y (50 *μ*g/kg OTA+0.4 mg/kg Se-Y)). Dosages of OTA were based on those reported by Solcan et al. [31], and Se-Y doses were based on those reported by Bakhshalinejad et al. [32].

### 2.4. Sample Preparation

Animal body weights were recorded after the beginning and the end of the experiment. Blood samples were collected from under the wings and then centrifuged (cryogenic centrifuge, Thermo Scientific, America) at 3000 × g at 4°C for 10 minutes to collect serum for analysis of serum creatinine, blood urea nitrogen, and uric acid. The chickens were euthanized and the kidneys were removed and weighed. The organ coefficients were calculated (organ wet weight percentage of the total body weight). The kidneys were fixed in 4% paraformaldehyde and stored for histopathological analysis. Samples from each group were homogenized in an appropriate amount of saline [tissue weight (g): saline volume (mL) = 1 : 9]. The remaining tissue was placed in a cryogenic vial and stored at -80°C for future analysis.

### 2.5. Histopathological Observation

The kidneys were removed from the 4% paraformaldehyde, embedded in paraffin, and sectioned. The kidney sections were then stained with H&E (slides were produced and analyzed by the China Seville Biotechnology Co., Ltd., Wuhan, China) for examination by light microscopy.

### 2.6. TUNEL Apoptosis Analysis

TUNEL Assay Kit (Roche, Basel, Switzerland) was used according to the manufacturer's instructions. The paraffin sections for TUNEL analysis were produced by the China Seville Biotechnology Co., Ltd., Wuhan, China.

### 2.7. Detection of Serum Creatinine, Blood Urea Nitrogen, and Uric Acid

An average of 5 mL of blood was collected from each chicken, and about 1.5 mL of serum was obtained. The appearance of serum was clear and transparent; only a few exhibited signs of hemolysis. Serum samples were analyzed for creatinine (CRE), blood urea nitrogen (BUN), and uric acid (UA) using commercially available kits (Nanjing Jiancheng Bioengineering Institute, Nanjing, China). The samples were prepared according the manufacturer's instructions.

### 2.8. Antioxidant Status

The kidneys were homogenized using a tissue homogenizer and centrifuged at 3500 × g at 4°C for 10 minutes. Fresh 10% tissue homogenate supernatants were used for BCA protein quantification (Nanjing Jiancheng Bioengineering Institute, Nanjing, China). Meanwhile, the antioxidant statuses of the kidneys were also assessed by analyzing the MDA and T-AOC levels using commercially available kits (Beijing Solarbio Science & Technology Co., Ltd., Beijing, China), as was T-GSH (Nanjing Jiancheng Bioengineering Institute, Nanjing, China). The antioxidant enzyme activities of T-SOD and CAT were measured using commercially available kits (Nanjing Jiancheng Bioengineering Institute, Nanjing, China). Each measurement was determined according to the instructions of the respective kit.

### 2.9. Analysis of Gene Expression

Total RNA was extracted from the kidneys using a Total RNA Extraction Kit (Vazyme, Nanjing, China) according to the manufacturer's instructions. Reverse transcription was performed using the PrimeScript™ RT Reagent Kit (Vazyme, Nanjing, China) and an ABI 7500 real-time PCR system according to the manufacturer's instructions. Expression levels of Nrf2, Keap1, HO-1, CAT, GSH-px, MnSOD, GLRX2, Bax, Caspase3, P53, AKT, PI3K, and Bcl-2 gene expressions in the kidneys were measured by qRT-PCR. The purity and concentration of total RNA were determined based on 260/280 nm OD values of single-stranded cDNAs that were synthesized by Sangon Biotech Co., Ltd. (Shanghai, China). Gene expression was calculated by the 2^-*ΔΔ*Ct^ method. All values were normalized to *β*-actin. The primer pairs used in this study are presented in Table 1.

### 2.10. Western Blot Analysis

Expressions of Nrf2, HO-1, CAT, MnSOD, Capase3, Bax, and Bcl-2 proteins in the kidneys of chickens were determined by western blot. The Protein Extraction Kit (Beyotime, Wuhan, China) was used to extract total proteins from the tissues. The BCA Protein Assay Kit (Solarbio, Beijing, China) was used to measure total protein content. The following antibodies were used to detect their respective proteins: anti-Caspase3 (1 : 1000, Abcam, Tokyo, Japan), anti-Bax (Immunoway, Suzhou, China), anti-HO-1, anti-Bcl-2, anti-Nrf2 (1 : 1000, Bioss, Beijing, China), anti-MnSOD (1 : 6000, Enzo Clinical Labs, Farmingdale, NY, USA), anti-CAT (1 : 700, Biorbyt, Cambridge, UK), anti-Actin (1 : 10000, Abcam, Tokyo, Japan). An HRP-labeled goat anti-rabbit IgG (1 : 10000, Jackson Immuno Research Labs, West Grove, PA, USA) was used as the secondary antibody. Relative band intensities were detected on a DNR Bio Imaging system by using the NcmECL Ultra method (Ncmbio, Suzhou, China). Relative intensities of these bands were normalized according to the *β*-actin.

### 2.11. Statistical Analysis

The SPSS 19.0 (IBM Corporation, Armonk, New York, USA) software was used to conduct all statistical tests. The results are presented as the mean ± standard error (*X* ± SE). Significant differences among the multiple groups were evaluated by one-way analysis of variance (ANOVA) with a post hoc test. Differences were considered significant at *p* < 0.05.

## 3. Results

### 3.1. Changes in the Body Weight and Organ Coefficient

**Figure 1:**
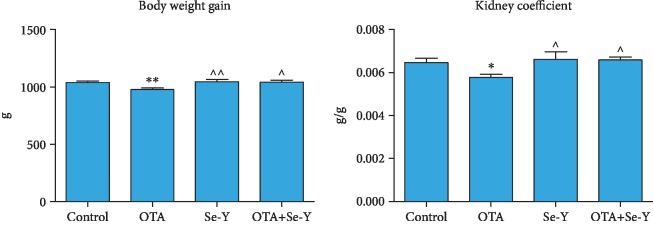
The body weight and the organ coefficients. The body weight and kidney organ coefficients in different groups. The organ coefficient = organ weight/body weight. Values represent the mean ± S.D.*n* = 7 chickens/group. Control: feeding the basal feed group; OTA: feeding the OTA-contaminated feed group; Se-Y: feeding the Se-Y feed group; OTA+Se-Y: feeding the OTA+Se-Y group. ^∗^*p* < 0.05, ^∗∗^*p* < 0.01 vs. control group. ^*p* < 0.05, ^^*p* < 0.01 vs. OTA group.

During the experiment, the health of the chickens was monitored daily. In the OTA group, transient diarrhea and dyspnea were observed. At the end of the experiment, the body weights and the organ coefficients of kidneys decreased significantly (*p* < 0.05) in the OTA group compared to the control group (Figure 1). The body weights and the organ coefficients of the kidneys were increased (*p* < 0.05) in the Se-Y-treated group than in the OTA group. In addition, these changes were improved in the OTA+Se-Y group compared to the OTA feed group. Meanwhile, there was no significant difference in the body weights and the organ coefficients between the control and Se-Y groups, indicating a lack of apparent detrimental effects of Se-Y supplementation. More importantly, there was no significant difference between the OTA+Se-Y and control groups.

### 3.2. Histopathological Changes in the Kidneys

**Figure 2:**
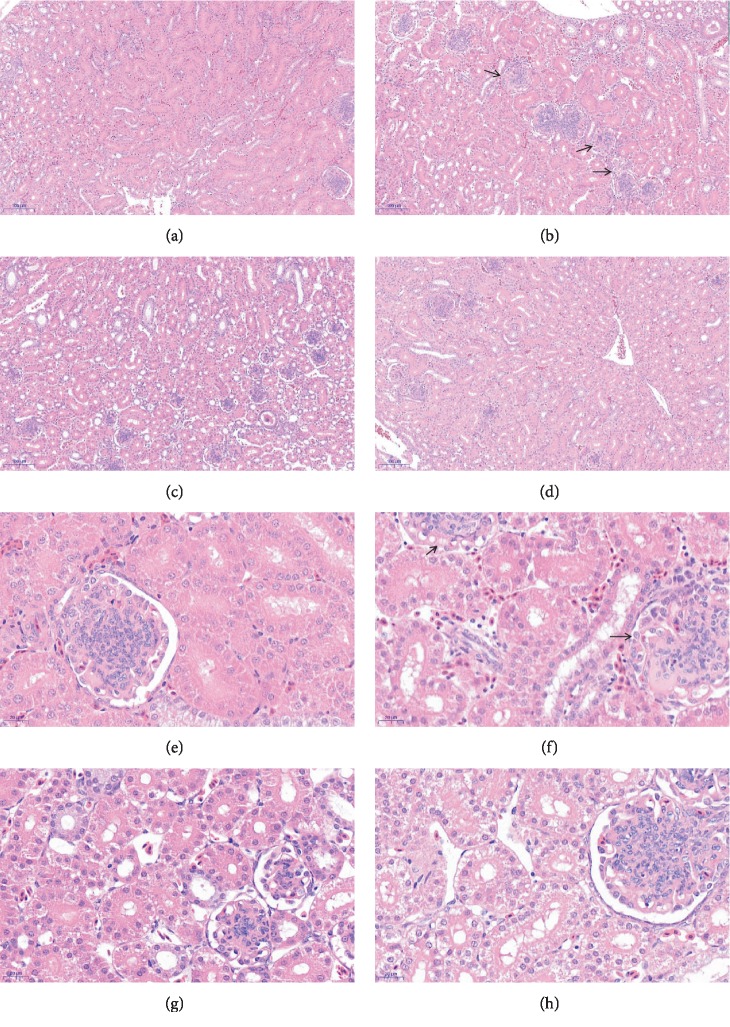
Detection of pathological changes in renal tissue by tissue section HE staining. Images were taken at a magnification of 100x (a–d) and 400x (e–h). (a, e) Control group, (b, f) OTA group, (c, g) Se-Y group, and (d, h) OTA+Se-Y group. *n* = 6 chickens/group. The arrows “→” indicate a pathological injury in the kidneys.

In the OTA group, histopathological examination showed glomerular swelling, filling the glomerular capsule, inflammatory cell infiltration, and hemorrhages in the kidneys (Figures 2(b) and 2(f)). Furthermore, the kidney sections from the OTA+Se-Y groups revealed minor pathomorphological changes (Figures 2(d) and 2(h)). These results indicate that Se-Y administration exerted a protective effect from OTA-induced kidney injury.

### 3.3. Analysis of Apoptosis by TUNEL

**Figure 3:**
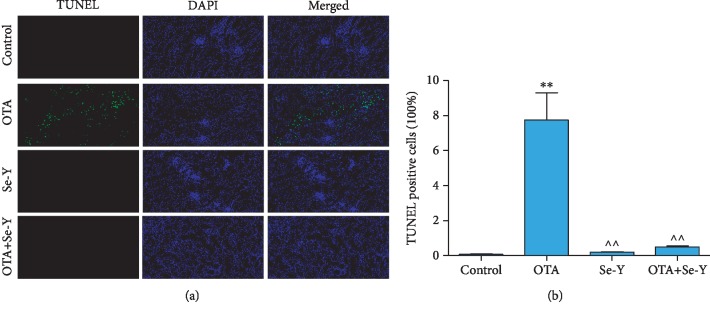
(a) TUNEL staining. Green fluorescence indicates TUNEL-positive cells. DAPI was used for nuclear staining (magnification 200x). (b) TUNEL-positive cells (100%). *n* = 6 chickens/group. ^∗^*p* < 0.05, ^∗∗^*p* < 0.01 vs. control group. ^*p* < 0.05, ^^*p* < 0.01 vs. OTA group.

As shown in Figure 3(a), the green fluorescence was particularly high in the OTA groups, which indicates that there was a large number of apoptotic cells in the kidney of chickens. As shown in Figure 3(b), the percentage of TUNEL-positive cells was increased (*p* < 0.05) in the OTA group than in the control group. More importantly, there was little green fluorescence in the control, Se-Y, and OTA+Se-Y groups, indicating that Se-Y had a certain inhibitory effect on apoptosis induced by OTA.

### 3.4. Changes in Serum Biochemical Parameters

**Figure 4:**
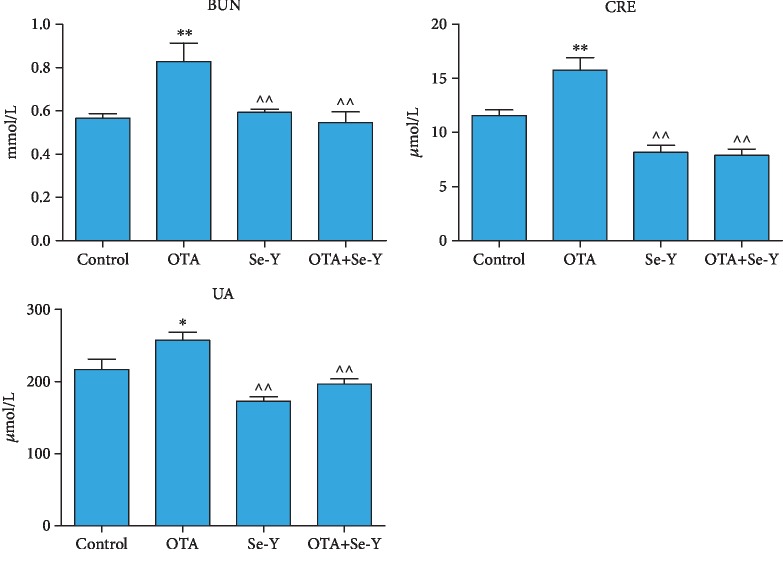
The detection results of serum biochemical parameters of chickens after the 21 days of diet treatment in each treatment group. *n* = 7 chickens/group. ^∗^*p* < 0.05, ^∗∗^*p* < 0.01 vs. control group. ^*p* < 0.05, ^^*p* < 0.01 vs. OTA group.

The changes of the serum biochemical parameters of chickens from each group are presented in Figure 4. The concentrations of CRE, BUN, and UA were higher (*p* < 0.05) in the OTA than the control group. Conversely, these markers significantly decreased in the Se-Y compared to the OTA group (*p* < 0.05). Furthermore, significant decreases in the levels of CRE, BUN, and UA were observed in the OTA+Se-Y compared to the OTA group (*p* < 0.05). Taken together, the data indicated that Se-Y was able to ameliorate the effects of OTA poisoning.

### 3.5. Analysis of the Oxidative Parameters of the Kidneys

**Figure 5:**
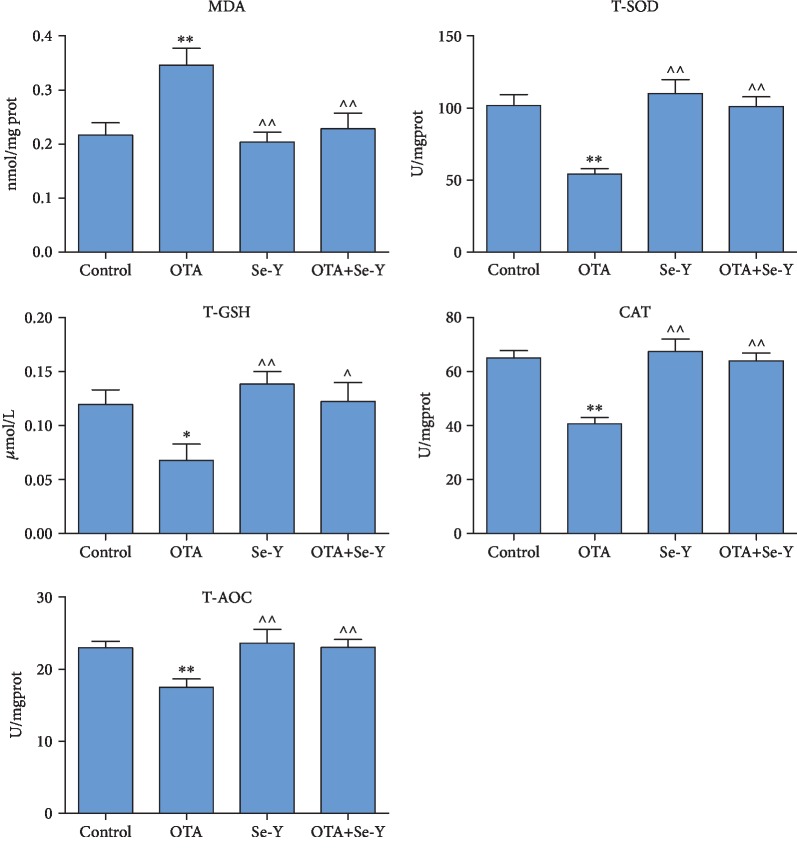
Oxidation and antioxidation parameters of renal tissues of chickens in each group were detected by an oxidation kit. *n* = 7 chickens/group. ^∗^*p* < 0.05, ^∗∗^*p* < 0.01 vs. control group. ^*p* < 0.05, ^^*p* < 0.01 vs. OTA group.

As presented in Figure 5, the oxidative parameters of kidney damage in the chickens were analyzed. The levels of MDA were significantly increased (*p* < 0.05), whereas the concentrations of T-GSH decreased (*p* < 0.05) in the kidneys of the OTA exposed versus the control chickens (Figure 5). However, relative to the OTA group, oral administration of Se-Y not only reversed the OTA-induced increase in MDA (*p* < 0.05) but also reversed the OTA-induced decrease of T-GSH (*p* < 0.05). The concentrations of the antioxidant enzymes T-SOD, T-AOC, and CAT were significantly decreased (*p* < 0.05) in the OTA versus the control group. In contrast, levels of the antioxidant enzymes increased in the OTA+Se-Y relative to the OTA group (*p* < 0.05).

In addition, analysis of the oxidative parameters of kidney damage failed to produce significant differences between the control, Se-Y, and OTA+Se-Y groups. The above results indicate that Se-Y exerted a nephron-protective effect from OTA-induced oxidative damage.

### 3.6. Effects of OTA on Gene Expression of the Nrf2/Keap1 and PI3K/AKT Signaling Pathways

**Figure 6:**
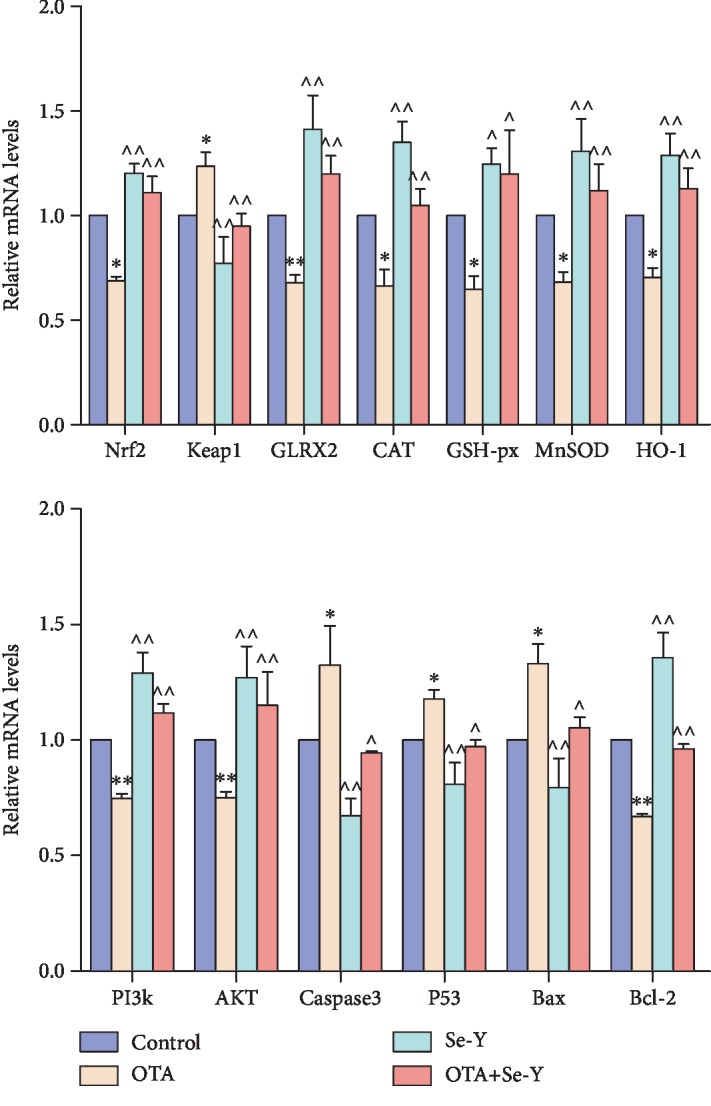
Effects of selenium yeast on the expression levels of genes involved in the Nrf2/Keap1 and PI3K/AKT signaling pathways that were induced by OTA in the chicken kidneys. *n* = 6 chickens/group. ^∗^*p* < 0.05, ^∗∗^*p* < 0.01 vs. control group. ^*p* < 0.05, ^^*p* < 0.01 vs. OTA group.

In order to observe the effects of low level (50 *μ*g/kg) OTA exposure on renal oxidative stress and apoptosis, the effects of OTA on gene expression of the Nrf2/Keap1 and PI3K/AKT signaling pathways were analyzed. The qRT-PCR analysis indicated that the Se-Y affects genes associated with the PI3K/AKT and Nrf2/Keap1 signaling pathways (Figure 6). The results showed that the levels of Nrf2, MnSOD, CAT, HO-1, GLRX2, GSH-px, AKT, PI3K, and Bcl-2 mRNA were downregulated (*p* < 0.05), while levels of Keap1, Bax, Caspase3, and P53 mRNA were increased (*p* < 0.05) in the OTA versus the control group. In addition, significant increases in Nrf2, MnSOD, CAT, HO-1, GLRX2, GSH-px, AKT, PI3K, and Bcl-2 mRNA levels (*p* < 0.05) along with significantly reduced expression of Keap1, Bax, Caspase3, and P53 (*p* < 0.05) were observed in the OTA+Se-Y group compared to the OTA group. More importantly, there was no significant difference between the OTA+Se-Y and control groups.

### 3.7. Effect of OTA Exposure on Protein Expression

**Figure 7:**
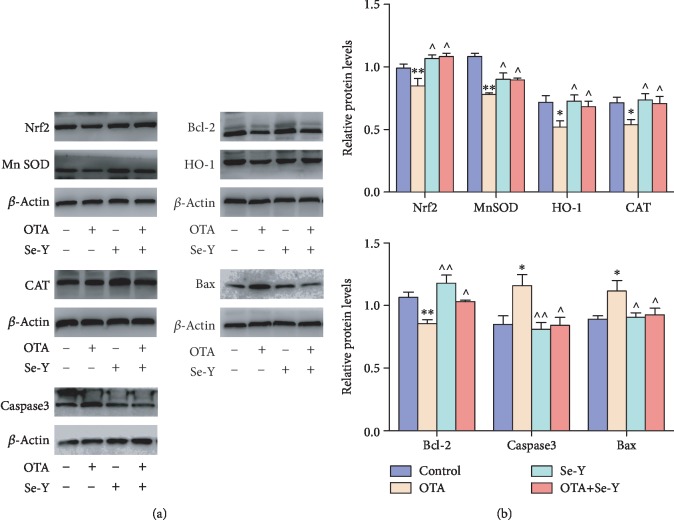
(a, b) Effects of selenium yeast on the expression levels of protein involved in the Nrf2/Keap1 and PI3K/AKT signaling pathways that were induced by OTA in the chicken kidneys. *n* = 6 chickens/group. ^∗^*p* < 0.05, ^∗∗^*p* < 0.01 vs. control group. ^*p* < 0.05, ^^*p* < 0.01 vs. OTA group.

To investigate whether exposure to OTA induces oxidative stress and apoptosis in the chicken kidneys, protein expressions of Nrf2, HO-1, CAT, MnSOD, Caspase3, Bax, and Bcl-2 were assessed by WB analysis (Figures 7(a) and 7(b)). Exposure to OTA downregulated the expression of Nrf2, HO-1, CAT, MnSOD, and Bcl-2 proteins examined (*p* < 0.05), while dietary supplementation of OTA-contaminated feed with Se-Y significantly ameliorated those effects. Taken together, our results further support the idea that Se-Y has a protective effect on OTA-induced toxicity in the kidneys of chickens.

**Figure 8:**
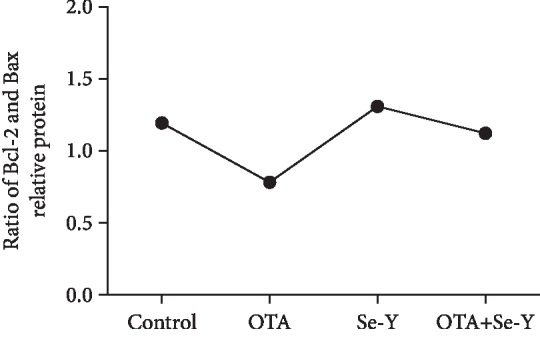
The ratio of Bcl-2 and Bax was used to indicate the apoptosis coefficient. *n* = 6 chickens/group.

The ratio of Bcl-2 and Bax has been proposed as a key factor in the regulation of apoptosis, and the low ratio of Bcl-2/Bax indicates increased apoptosis. As shown in Figure 8, a lower ratio of Bcl-2/Bax was observed in the OTA group compared to control group. However, there were no significant changes in the Bcl-2/Bax ratio between the Se-Y and OTA+Se-Y groups.

These results indicate that administering the Se-Y exerted a protective effect from OTA-induced apoptosis and oxidative stress in the chicken's kidneys.

## 4. Discussion

In recent years, much research has sought effective dietary supplements to aid in detoxification that are eco-friendly as well. Anti-OTA dietary supplements are of great interest due to the extensive toxicity of OTA in addition to the fact that these supplements do not cause significant feed safety problems without compromising nutrient loss. However, there have not been any reports on the protective efficacy of Se-Y against OTA-mediated oxidative damage at the time of publication.

Because of its chemical stability and widespread distribution, OTA contamination of livestock feed is all but avoidable. The nephrotoxicity of OTA has been well established not only in fish, mice, chicken, and pigs, but in humans as well [33–37]. At low doses, OTA reduced the growth rate, kidney coefficient, feed consumption, and induced degenerative changes of kidney epithelium [35, 38]. In the present study, it was observed that the body weights and kidney coefficients in OTA-exposed chickens decreased significantly relative to the control group (*p* < 0.05). Histopathological changes observed here that were associated with OTA exposure included swelling of the kidney and caused glomerular swelling, filling the glomerular capsule, inflammatory cell infiltration, and hemorrhages in the kidneys, which were consistent with previous reports [39]. Oxidative stress occurs when the body is subjected to various noxious stimuli, and high activity molecules such as reactive oxygen (ROS) and reactive nitrogen (RNS) free radicals are produced [40]. It has been reported that OTA can induce the production of ROS [41, 42]. The MDA, T-GSH, SOD, CAT, and T-AOC are important enzymes of the endogenous antioxidant defense system, which play an important role in the maintenance of intracellular redox balance. The data presented here show decreases of enzymatic activities for T-SOD, T-AOC, CAT, and T-GSH, in the OTA versus the control group. In addition, an increase of the kidney MDA levels post-OTA exposure was observed, suggesting free radicals are responsible for the pathologic changes. In addition, oral administration of Se-Y significantly changed the tissue enzymology induced by OTA. Thus, the antioxidant effect of Se-Y seems to play an important role in the protection of the kidneys exposed to OTA.

It has been reported in previous studies that OTA, through the activation of the PI3K/AKT signaling pathways, reduces the viability of testicular and porcine granulosa cells [43, 44]. *In vivo* experiments have demonstrated the ability of Se to improve Caspase3 and P53 levels [45]. To this end, expression of apoptosis-related genes within the kidneys was analyzed. The data indicated that exposure to OTA through feed leads to apoptosis, similar to observations made in other studies [46, 47]. Some other studies have reported that OTA-contaminated diets had a significant effect on LDH levels, Caspase3 activities, AKT levels, and numbers of apoptotic nuclei [48, 49]. Here, it was demonstrated that mRNA expressions of Bax, P53, and Caspase3 were increased in the OTA-exposed kidneys, while levels of PI3K, AKT, and Bcl-2 decreased. Furthermore, Se-Y significantly improved expression of apoptosis-related genes following exposure to OTA. These results are consistent with others reporting that Se-Y exhibits antiapoptosis effects *in vivo* and *in vitro* [19]. Here, we report that Se-Y-mediated protection of the kidneys against OTA toxicity occurred through selective repression of proapoptotic genes.

Induction of oxidative stress by OTA has been reported in several studies [50, 51]. Selenium has been shown to antagonize the cytotoxicity and oxidative stress injuries caused by ochratoxin. Reszka et al. [52] also demonstrated that plasma Se levels were negatively correlated with Keap1 levels and positively correlated with Nrf2 levels. Those findings were similar to those reported here, which showed that supplementation with Se-Y to feed contaminated with OTA significantly improved Keap1 and Nrf2 levels in the chicken kidneys. Loboda et al. [53] reported that Nrf2 deficiency exacerbates OTA-induced toxicity in vitro and in vivo. Furthermore, Nrf2 and its target genes, such as MnSOD, CAT, GSH-px, HO-1, and GLRX2, are crucial components of the endogenous redox system [54, 55]. Noteworthy, Nrf2 and its target gene, HO-1, were markedly decreased following OTA exposure in renal proximal tubular epithelial cells [56, 57]. In the present study, it was observed that Se-Y activated Nrf2, resulting in enhanced expression of Nrf2, MnSOD, CAT, GSH-px, HO-1, and GLRX2, leading to improved oxidative stress resistance. These changes resulted in improvement of the redox balance and enhanced the resistance of the chicken kidneys to oxidative stress induced by OTA. It is well known that these proteins exert cytoprotective effects to oxidative stress [45, 58–61]. The data presented here indicate that Se-Y could improve OTA-induced oxidative damage through activation of the Nrf2 signaling pathway in the kidneys of chickens.

## 5. Conclusions

The data presented here indicate that exposure to OTA results in oxidative damage and apoptosis in the chicken kidneys. Conversely, Se-Y protects the kidneys from OTA-induced damage through the modulation of the Nrf2/Keap1 and PI3K/AKT signaling pathways. Moreover, the findings reported here suggest a mechanism through which Se-Y exerts its antioxidant and antiapoptotic effects. Furthermore, this study provides additional evidence favoring the use of Se-Y as a natural agent for the prevention of OTA-induced oxidative damage and apoptosis in the kidneys of chickens.

## Figures and Tables

**Table 1:** The primers sequence of the target genes.

Primer	Sequence	Amplicon size (bp)
R-Nrf2-F:	5′ CATAGAGCAAGTTTGGGAAGAG 3′	105 bp
R-Nrf2-R:	5′ GTTTCAGGGCTCGTGATTGT 3′	
R-Keap1-F:	5′ ACTTCGCTGAGGTCTCCAAG 3′	142 bp
R-Keap1-R:	5′ CAGTCGTACTGCACCCAGTT 3′	
R-GSH-Px-F:	5′ CCAATTCGGGCACCAGGAGAA 3′	157 bp
R-GSH-Px-R:	5′ CTCTCTCAGGAAGGCGAACAG 3′	
R-MnSOD-F:	5′ AAGGAGCAGGGACGTCTACA 3′	82 bp
R-MnSOD-R:	5′ CCAGCAATGGAATGAGACCTGT 3′	
R-Hmox1-F:	5′ ACGTCGTTGGCAAGAAGCATCC 3′	191 bp
R-Hmox1-R:	5′ TTGAACTTGGTGGCGTTGGAGAC 3′	
R-CAT-F:	5′ TGTCTCAGGTGCGAGACTTC 3′	156 bp
R-CAT-R:	5′ GTGCGCCATAGTCAGGATGA 3′	
R-GLRX2-F:	5′ ACGGAAGCCAGATCCAAGAC 3′	151 bp
R-GLRX2-R:	5′ GTAGCACCTCCAACAAAAGACC 3′	
R-Bax-F:	5′ GTGATGGCATGGGACATAGCTC 3′	91 bp
R-Bax-R:	5′ TGGCGTAGACCTTGCGGATAA 3′	
R-Bcl-2-F:	5′ ATCGTCGCCTTCTTCGAGTT 3′	151 bp
R-Bcl-2-R:	5′ GTAGCACCTCCAACAAAAGA 3′	
R-PI3K-F:	5′ TACATTCTTGGGCTCCTT 3′	170 bp
R-PI3K-R:	5′ AGTGCGTGGAAATCTAAT 3′	
R-AKT-F:	5′ AGTGCGTGGAAATCTAAT 3′	146 bp
R-AKT-R:	5′ ATAATGACTATGGTCGTGC 3′	
R-CASP3-F:	5′ AAGCGAAGCAGTTTTGTTTGTG 3′	128 bp
R-CASP3-R:	5′ GCTAGACTTCTGCACTTGTCACCTC 3′	
R-p53-F:	5′ AAGCGAAGCAGTTTTGTTTGTG 3′	157 bp
R-p53-R:	5′ GCTAGACTTCTGCACTTGTCACCTC 3′	
R-*β*-actin-F:	5′ AGGAGAAGCTGTGCTACGTC 3′	183 bp
R-*β*-actin-R:	5′ TACCACAGGACTCCATACCCAA 3′	

## Data Availability

The data used to support the findings of this study are included within the article.
